# Virtual Contrast for Coronary Vessels Based on Level Set Generated Subvoxel Accurate Centerlines

**DOI:** 10.1155/IJBI/2006/94025

**Published:** 2006-09-03

**Authors:** Ingmar Bitter, Robert Van Uitert, Ivo Wolf, Efstathia Tzatha, Ahmed M Gharib, Ronald Summers, Hans-Peter Meinzer, Roderic Pettigrew

**Affiliations:** ^1^ Diagnostic Radiology Department, Clinical Center, National Institutes of Health, Bethesda, MD 20892-1182, USA; ^2^ Division of Medical and Biological Informatics, German Cancer Research Center, Heidelberg 69120, Germany; ^3^ National Heart Lung and Blood Institute, National Institutes of Health, Bethesda, MD 20892-2281, USA

## Abstract

We present a tool for tracking coronary vessels in MRI scans of the human heart to aid in the screening of heart diseases. The vessels are identified through a single click inside each vessel present in a standard orthogonal view. The vessel identification results from a series of computational steps including eigenvalue analysis of the Hessian of the MRI image followed by a level set-based extraction of the vessel centerline. All identified vessels are highlighted using a virtual contrast agent and displayed simultaneously in a spherical curved reformation view. In cases of over segmentation, the vessel traces can be shortened by a click on each vessel end point. Intermediate analysis results of the vessel computation steps can be displayed as well. We successfully validated the tool on 40 MRI scans demonstrating accuracy and significant time savings over manual vessel tracing.


Hindawi Publishing Corporation
International Journal of Biomedical Imaging
Volume 2006, Article ID 94025, Pages 1–8
DOI 10.1155/IJBI/2006/94025

### Virtual Contrast for Coronary Vessels Based on Level Set Generated Subvoxel Accurate Centerlines

**Ingmar Bitter,[1] Robert Van Uitert,[1] Ivo Wolf,[2] Efstathia Tzatha,[3] Ahmed M. Gharib,[3] Ronald Summers,[1] Hans-Peter Meinzer,[2] and Roderic Pettigrew[3]**

[1] *Diagnostic Radiology Department, Clinical Center, National Institutes of Health, Bethesda, MD 20892-1182, USA*
[2] *Division of Medical and Biological Informatics, German Cancer Research Center, Heidelberg 69120, Germany*
[3] *National Heart Lung and Blood Institute, National Institutes of Health, Bethesda, MD 20892-2281, USA*

Received 31 January 2006; Revised 30 May 2006; Accepted 6 June 2006

We present a tool for tracking coronary vessels in MRI scans of the human heart to aid in the screening of heart diseases. The vessels are identified through a single click inside each vessel present in a standard orthogonal view. The vessel identification results from a series of computational steps including eigenvalue analysis of the Hessian of the MRI image followed by a level set-based extraction of the vessel centerline. All identified vessels are highlighted using a virtual contrast agent and displayed simultaneously in a spherical curved reformation view. In cases of over segmentation, the vessel traces can be shortened by a click on each vessel end point. Intermediate analysis results of the vessel computation steps can be displayed as well. We successfully validated the tool on 40 MRI scans demonstrating accuracy and significant time savings over manual vessel tracing.

Copyright © 2006 Ingmar Bitter et al. This is an open access article distributed under the Creative Commons Attribution License, which permits unrestricted use, distribution, and reproduction in any medium, provided the original work is properly cited.

### 1. INTRODUCTION

Coronary artery disease (CAD) is one of the leading causes of mortality and morbidity in the USA and other industrialized nations [1]. Although conventional cardiac angiography remains the "gold standard" for the evaluation of CAD, it is an invasive procedure that is associated with morbidity (1.5%) and mortality (0.15%) risks [2]. Coronary CT angiography (CTA) is emerging as a promising noninvasive alternative [3]. However, this technology requires patient exposure to substantial amounts of radiation [4] and potentially nephrotoxic contrast agents as in conventional angiography. As a result, coronary magnetic resonance angiography (CMRA) provides a more patient friendly option for CAD assessment [5] without the use of contrast agents and radiation. Unfortunately, currently the MRI image signal-to-noise ratios and the maximally achievable resolution are not as high as for CTA. This complicates the process of identifying the vessels and MRA targeted vessel segmentation and analysis tools usually fail. Thus, the common solution is still time consuming, manual vessel tracing. Our research presents an MRI coronary identification software tool that is able to track and intuitively display the MRI data along all three main coronary vessels. A typical screenshot of the software is presented in Figure 1.

A core component of the system is the computation of a centerline for each vessel. As the vessels are only a few voxels thick, it is important to compute the vessel centerlines at subvoxel accuracy. They must also be inherently smooth to yield a smooth display of the vessel in the spherical curved reformation view.

The next section describes the technical background of our methods followed by related work and a description of the novel methods used in our system. We conclude with showing and discussing results acquired with our software.

### 2. BACKGROUND

Many automatic and semiautomatic skeletonization techniques compute the centerline of an object on the voxel grid with optional subsequent smoothing [6–16]. These discrete centerline solutions are inappropriate for vessels that are only a few voxels thick. Subsequent smoothing may displace the centerline from the vessel center and is thus inappropriate as well. Another method [17] computes the centerline as a minimum cost B-spline. This delivers an inherently smooth centerline, but is computationally expensive due to the explicit global optimization. The same holds for [18], which iteratively computes a globally optimized NURBS curve that locally minimizes the vessel cross sections perpendicular to

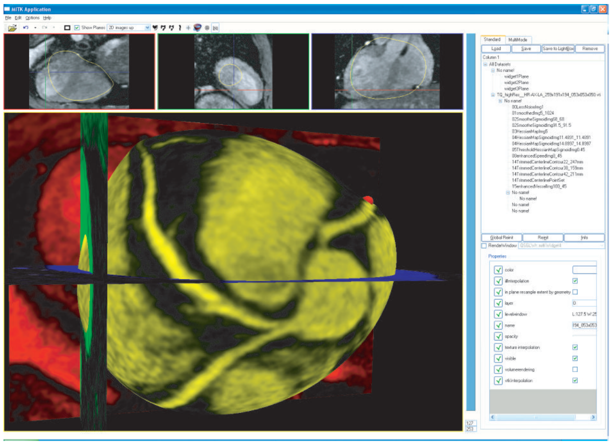

Figure 1: A screenshot of the MRI coronary vessel tracking tool with axial (red), sagittal (green), coronal (blue), and spherically fitted "thin plate spline" (yellow) 3D views showing all three main coronaries virtually enhanced.

the curve. In contrast, our prior centerline algorithm efficiently and automatically extracts a smooth, continuous centerline directly at subvoxel precision [19]. Our algorithm is based on level sets. Level set methods evolve an isosurface in the direction of the surface normal [20]. In its general form the evolution speed can depend on position, normal direction, curvature, and shape; and the isosurface can cross over the same point multiple times. In our centerline method the evolution speed is always positive and depends only on position. Hence, its boundary front moves only outwards. With these restrictions the isosurface can be represented by an Eikonal equation:

$$|\nabla T|F = 1, \quad T = 0 \text{ on } \Gamma, \tag{1}$$

where $T$ is the arrival time function, $F$ is the speed of evolution function, and $\Gamma$ is the initial isosurface at time zero.

An efficient method to numerically evaluate the solution to the Eikonal equation is the fast marching method [20]. It processes the voxels in a sorted order based on increasing values of $T$. The fast marching method calculates a time crossing map, which indicates for each pixel how much time it would take for the level set front to arrive at the pixel location. The evolution only needs to be computed on a rectilinear grid. However, values at nongrid locations can be interpolated from these grid positions properly to simulate the true propagation value.

Several other centerline methods based on level sets have been previously presented [10–12]. One approach [10] computes centerlines by first detecting medial axis points at the locations where the level set fronts collide and form sharp discontinuities. However, the level sets are only computed on two dimensional cross-sections of the three dimensional data, which are not identical to the 3D discontinuities. Next, the algorithm performs topological thinning and filling in of gaps with voxels along straight lines which may not result in positions on the skeleton. Other methods [11, 12] make use of the full 3D data in its level set propagation and guarantee a minimum cost, discrete solution, but as pointed out before, do not extract the centerline with subvoxel accuracy. In addition, algorithms [6–10] require a segmentation of the vessel as input. In our images it is very difficult to segment the vessels accurately and completely. Hence, we were looking for an algorithm that does not require a segmented vessel as input. An algorithm that is subvoxel accurate and does not require vessel segmentation is presented in [21]. It directly traverses the centerline along ridges in a Hessian medialness measure, but it performs a sequence of local greedy decisions that do not guarantee a globally optimal solution. The methods in [11–17, 21] use Hessian matrix analysis to track a vessel with having to segment its boundaries first. Computing the Hessian at different scales proved to be beneficial for vessels varying greatly in thickness, but was not necessary for our data. Table 1 lists the prior methods and classifies them according to the main algorithm ideas. In [22], 94 vessel extraction algorithms are compared: only 50% of them do compute an explicit centerline, only one uses level set methods, but not for centerline tracking and only one uses Hessian eigenvalues, but not in combination with level sets. Our proposed algorithm combines the benefits of the previous methods without their shortcomings. It can find minimum cost, subvoxel accurate centerlines of thin vessels without the need of first segmenting them.

### 2.1. *Subvoxel accurate centerline algorithm*

The most closely related prior algorithm is our input segmentation dependent, subvoxel accurate centerline algorithm [19]. It uses a level set segmentation of the vessel to obtain a subvoxel accurate surface and a Euclidean distance transform of the object. This distance transform is then used as a speed image in a fast marching level set method with propagation starting at the global maximum point of the distance transform. The fast marching method propagation is augmented to calculate the geodesic distance in addition to the time crossing map. The furthest geodesic point from the global maximum point is used as the start point of the vessel centerline and the remaining points of the centerline are determined by performing a gradient descent on the time crossing map with a subvoxel step size.

The algorithm presented in this paper is an extension of this previous method, which handles the absence of vessel segmentation and improves upon the computation speed of the level set propagation.

### 2.2. *Vessel enhancement*

In order to track thin vessels without an explicit representation, we found it necessary to process the MRI images using vessel enhancing image filters. Given the eigenvalues $\lambda_3 \le \lambda_2 \le \lambda_1$ of the $3 \times 3$ Hessian matrix for each 3D image pixel, it is possible to compute a likelihood of the pixel being part of a linear structure [23, 24]. This measure, which we

Table 1: Comparison of ideas used in various vessel centerline computation methods. "+" stands for the best idea within a group, "0" for average, and "−" for the least desirable idea in a group.

| Vessel centerline computation reference | [6–10] | [11] | [17] | [18] | [12] | [13] | [14, 15] | [16] | [19] | This paper |
|---|---|---|---|---|---|---|---|---|---|---|
| 2D | | | | | | − | − | | | |
| 2D and 3D | + | + | + | + | + | | | + | + | + |
| Prior vessel segmentation required | − | | | | | | | | − | |
| Vesselness from intensity only | | − | | | | | | | | |
| Vesselness from Hessian eigenvalues | | | + | + | + | + | + | + | | + |
| Path cost from segmentation distance map | − | | | | | | | | − | |
| Path cost from vesselness | | 0 | | | 0 | | 0 | | | 0 |
| Path cost from multiscale vesselness | | | + | | | + | + | + | | |
| Discrete cost propagation | − | | | | | − | − | − | | |
| Level set wave cost propagation | | + | | | + | | | | + | + |
| Centerline extraction as minimum cost B-spline | | | 0 | | | | | | | |
| Centerline as minimum vessel cross-section NURBS | | | | 0 | | | | | | |
| Discrete centerline, optional post smoothing | − | − | | | − | − | − | − | | |
| Smooth centerline (wave gradient decent) | | | | | | | | | + | + |
| Computation only for the most obvious path | | | | | | | | | | + |

call *vesselness* ($v$), is defined as

$$v = \begin{cases} |\lambda_3| \cdot \psi(\lambda_2;\lambda_3) \cdot \omega(\lambda_1;\lambda_2) & \forall \lambda_3 \leq \lambda_2 < \lambda_1, \\ 0 & \text{otherwise} \end{cases} \quad (2)$$

with

$$\psi(\lambda_2;\lambda_3) = \begin{cases} \left(\dfrac{\lambda_2}{\lambda_3}\right)^{\gamma} & \forall \lambda_3 \leq \lambda_2 < 0, \\ 0 & \text{otherwise}, \end{cases} \quad (3)$$

$$\omega(\lambda_1;\lambda_2) = \begin{cases} \left(1 + \dfrac{\lambda_1}{|\lambda_2|}\right)^{\gamma} & \forall \lambda_2 \leq \lambda_1 < 0, \\ \left(1 + \frac{\alpha\lambda_1}{|\lambda_2|}\right)^{\gamma} & \forall \dfrac{|\lambda_2|}{\alpha} > \lambda_1 > 0, \\ 0 & \text{otherwise} \end{cases} \quad (4)$$

and $\alpha = 1/4$ and $\gamma = 1/2$.

This *vesselness* $v$ has been used before to improve visualizations of linear structures [23, 24], but we are using it to assist in vessel tracking. However, other similar equations have been used for vessel identification before [25].

### 2.3. Curved reformation vessel view

A good vessel centerline can be used to create a curved reformation vessel view. One such method is to stretch the vessel and display its surroundings with as little distortion as possible [26]; however this is not appropriate for a view that is supposed to include multiple vessels. The "soap bubble" method [27] allows projection of multiple vessels to a plane, preserving the relationship of the vessels to each other, but for roughly spherically arranged vessels, the projected vessels may overlap or surrounding tissue may be severely distorted. We use the spherical curved reformation method [28], which eliminates the problems of the "soap bubble" method for the specific case of coronary vessels. It achieves this through projecting a spherical approximation of the heart onto the vessels with little distortion, followed by a standard globe unrolling onto a rectangular view as done for any world map. The minimal distortion is the consequence of minimizing the energy of a "thin plate spline" being fit to the vessel points [28].

### 3. METHODS

While the system is designed to look at all vessels at once, processing is done one vessel at a time. The user identifies each vessel initially by clicking on one landmark point for each vessel.

### 3.1. Vessel centerline computation

The vessel centerline computation results from a series of computational steps (Figure 2). Initially, noise removal is performed on the MRI data by using edge preserving anisotropic diffusion filtering. Next, the intensities are normalized through a sigmoid window whose parameters are

(1) Remove noise
(2) Place landmark A inside vessel
(3) Normalize intensities
(4) Compute vesselness
(5) Sigmoid vesselness to speed image
(6) Propagate wave to spearhead start
(7) Propagate spearhead wave
(8) Autocreate landmark B at end
(9) Back trace partial centerline to A
(10) Intensify speed image on centerline
(11) Propagate wave to spearhead start
(12) Propagate spearhead wave
(13) Autocreate landmark C at end
(14) Back trace full centerline to B
(15) Create spherical reformation
(16) Crop vessel end points to D and E
(17) Repeat for other vessels

Figure 2: Steps of the algorithm needed to track and display vessels.

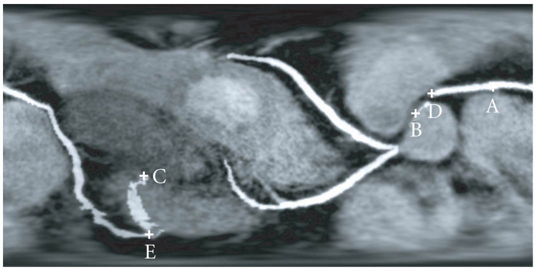

Figure 3: Landmark A is initially manually placed inside the vessel. Landmarks B and C are automatically placed by the algorithm. Vessel end points D and E are manual corrections of points B and C which are placed on the curved view without the need to scroll through multiple images.

determined from the MRI intensity I at the first landmark point (A in Figure 3) which must be inside the vessel and is assumed to be the maximum intensity in the local neighborhood. The width of the sigmoid window is equal to I and the center is at I/2. Subsequently the vesselness, $v$, is computed for each pixel in the image by (2) from the Hessian matrix of the image. The partial derivatives that form the Hessian matrix are a result of convolving the smoothed MRI image with the derivatives of a Gaussian with $3\sigma$ covering 2 mm, which is the median of the expected vessel diameter. This vesselness map is then normalized using a sigmoid window. Again the window parameters are relative to the landmark point (width = õ, center = õ/2), which maximizes the contrast in the transition region. Values in the normalized vesselness map are clamped to zero if they are less than 33% of the maximum value. The clamp threshold and $\sigma$ were determined empirically for a single dataset and applied for all others.

This resulting *vesselness* map (middle image in Figure 4) is used as a speed function for a fast marching level set method that starts at the initial vessel landmark point A. However, instead of computing the fast marching through the complete image or at least until the entire vessel is covered (as in [11]), the computations are stopped when a point 1 cm from the landmark point is reached. Due to the nature of the *vesselness* computation, the highest speed values are found in the center of the vessel, and thus the point first reached at 1 cm distance (larger than the vessel radius) must be central to the vessel. This point is then defined as the "spearhead point." The fast marching method is now continued, but only newly discovered points that are within 1 cm of the "spearhead point" are allowed to be added to the evolving surface of the fast marching method. Each time a new furthest geodesic distance point is found, the "spearhead point" is updated. Consequently, only a small band of voxels along the vessel is involved in the computation. When the modified fast marching method has processed all of the points in the connected object, the final "spearhead point" is the most distant trackable vessel point. This becomes the second, automatic landmark point (B in Figure 3). The steepest gradient decent from the second to the first landmark point yields a partial vessel centerline. This centerline is not based on local greedy decisions, but is the minimum cost path with respect to the given *vesselness* speed image. Next, the speed image is intensified along this partial vessel. With the updated speed image, the above described fast marching method is started from the second automatic landmark. Again, only a small band of voxels along the vessel are involved in the computation. When the second fast marching method algorithm completes, the resulting "spearhead point" becomes the third, automatic landmark point (C in Figure 3). Intensifying the speed image along the first partial centerline is necessary to guarantee that the initial path of the second fast marching does not detour into a vessel branch. Finally, the steepest gradient decent from the third (C) to the second (B) landmark point yields the complete vessel centerline (Figure 5).

Once the centerline is computed, a virtual contrast dataset can be created. The virtual contrast dataset has the original data as its basis, but each pixel is intensified that is within 2 mm (expected vessel radius) of the computed centerline and also has at least 33% of the maximal vesselness intensity in the initial speed image.

The above process can be repeated for each of the desired vessels. After the centerline for each vessel has been computed, a new spherical curved reformation can be generated from all current vessel centerlines. The length of each of the vessels is displayed on the graphical user interface. In order to be able to bridge areas of stenosis or low signal, short (< 1 cm) sections of low *vesselness* (below 33% of the maximum intensity value) are allowed, as long as the tracking can be continued with more obvious *vesselness* pixels after the gap. Unfortunately, this sometimes also causes the tracking to go beyond the vessel ends into other nearby vessels or to jump onto the edge of the heart, which may not be totally suppressed in the speed image. In this case, the curved reformation view can be used

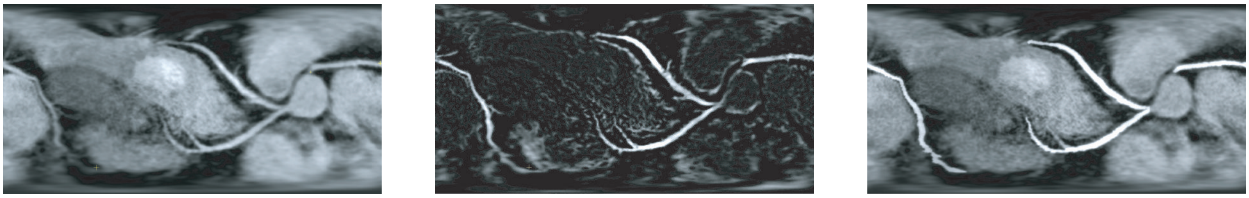

(a) (b) (c)

Figure 4: Three key steps along the processing pipeline: smoothed image, vesselness map speed image, and virtual contrast image.

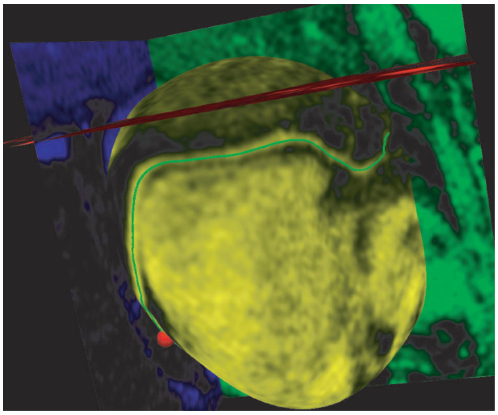

Figure 5: Spherical reformation with superimposed vessel centerline.

to allow the user to manually relocate the second and/or third landmark point to the desired vessel endpoint(s) (D and E in Figure 2). The system then adjusts the centerline to only cover the vessel between these updated vessel end points. System validation was based on visual assessment of completeness of the tracked vessels, partial success was defined as a section of the vessel being visible in the scan, but not tracked. The scanning protocols used for the validation were (A) standard imaging parameter, (B) shortened acquisition window, (C) isotropic acquisition voxel resolution, (D) short-axis plane aligned with the right coronary artery.

### 4. RESULTS

The algorithm presented was validated on 40 MRI cardiac scans with volume sizes of $512 \times 512 \times 100$ to $512 \times 512 \times 300$ containing $0.5 \times 0.5$ mm images with 0.5 to 1 mm spacing in the $z$-direction. The data came from 10 volunteers scanned each with four scanning protocols. The right coronary artery (RCA) was found completely for 90% of the volunteers on two protocols (A, B), for the other protocols 80% (B) and 70% (D) of the MRI scans had completely tracked vessels. For the incomplete scans it was possible to complete them by treating the missed vessel section as a new vessel. Figure 3 shows some intermediate and final results.

Completing the interactive part of the vessel tracking was accomplished within one minute. On a professional medical image analysis workstation a trained radiologist took 2.5 minutes to hand segment a vessel via coarse contours on every third slice that were then interpolated by the system.

### 5. DISCUSSION

The results in Figures 1, 3, and 5 show spherical curved reformations of the three main coronary arteries. In this example for two arteries joined near the aorta a single landmark was sufficient, but for the third both endpoints needed to be corrected. In either case, the user interaction time required is minimal when compared to manual vessel tracking.

All numerical parameters listed in this algorithm degrade gracefully. A 10% change of the parameter value has only a small impact on the final result, but doubling or halving it usually significantly shortens the identification of vessel segment.

The novelty of this research is two fold. First, it lies in the creation of a time saving tool that combines the idea of semi-automatic tracking with spherical curved reformation. Second, it improves over prior work on the method of finding the vessel centerline. Due to the low signal-to-noise ratio on the MRI input images, the *vesselness* map is a network of mutually connected vessels and pseudovessels. Following all branches as done in [10] frequently results in automatically found vessel end landmarks that are very far from the intended vessel end. The restriction of expanding the fast marching only near the "spearhead point" allows for a much more intuitive behavior of the algorithm. The manual clipping of the traced path to only the portion within the vessels is easily performed on the spherical curved reformation view and no scrolling through slices is needed.

### 6. CONCLUSIONS

This paper has presented a semi-automatic algorithm for determining centerlines of the main coronary vessels and its application to creating virtual contrast enhanced MRI scans that are displayed in an intuitive spherical curved reformation view. The method can track vessels even in the presence of low signal-to-noise ratios, is subvoxel accurate, and is more computationally efficient than previous methods.

### REFERENCES

[1] AHA, "Heart Disease and Stroke Statistics-2005 Update," 2005, http://www.americanheart.org/.

[2] L. W. Johnson, R. Krone, M. J. Cowley, et al., "Cardiac catheterization 1991: a report of the Registry of the Society for Cardiac Angiography and Interventions (SCA and I)," *Catheterization and Cardiovascular Diagnosis*, vol. 28, no. 3, pp. 219–220, 1993.

[3] S. Schroeder, A. Kuettner, T. Beck, et al., "Usefulness of noninvasive MSCT coronary angiography as first-line imaging technique in patients with chest pain: initial clinical experience," *International Journal of Cardiology*, vol. 102, no. 3, pp. 469–475, 2005.

[4] C. H. McCollough, "Patient dose in cardiac computed tomography," *Herz*, vol. 28, no. 1, pp. 1–6, 2003.

[5] W. Y. Kim, P. G. Danias, M. Stuber, et al., "Coronary magnetic resonance angiography for the detection of coronary stenoses," *New England Journal of Medicine*, vol. 345, no. 26, pp. 1863–1869, 2001.

[6] I. Bitter, M. Sato, M. Bender, K. T. McDonnell, A. Kaufman, and M. Wan, "CEASAR: a smooth, accurate and robust centerline extraction algorithm," in *Proceedings of the IEEE Visualization Conference*, pp. 45–52, Salt Lake City, Utah, USA, October 2000.

[7] I. Bitter, A. E. Kaufman, and M. Sato, "Penalized-distance volumetric skeleton algorithm," *IEEE Transactions on Visualization and Computer Graphics*, vol. 7, no. 3, pp. 195–206, 2001.

[8] Y. Zhou and A. W. Toga, "Efficient skeletonization of volumetric objects," *IEEE Transactions on Visualization and Computer Graphics*, vol. 5, no. 3, pp. 196–209, 1999.

[9] D. Chen, B. Li, Z. Liang, M. Wan, A. Kaufman, and M. Wax, "Tree-branch searching, multiresolution approach to skeletonization for virtual endoscopy," in *Medical Imaging 2000: Image Processing*, vol. 3979 of *Proceedings of SPIE*, pp. 726–734, San Diego, Calif, USA, February 2000.

[10] A. Telea and A. Vilanova, "A robust level-set algorithm for centerline extraction," in *Symposium on Visualization (VisSym '03)*, pp. 185–194, Grenoble, France, May 2003.

[11] T. Deschamps and L. D. Cohen, "Fast extraction of minimal paths in 3D images and applications to virtual endoscopy," *Medical Image Analysis*, vol. 5, no. 4, pp. 281–299, 2001.

[12] O. Wink, A. F. Frangi, B. Verdonck, M. A. Viergever, and W. J. Niessen, "3D MRA coronary axis determination using a minimum cost path approach," *Magnetic Resonance in Medicine*, vol. 47, no. 6, pp. 1169–1175, 2002.

[13] O. Wink, W. J. Niessen, and M. A. Viergever, "Multiscale vessel tracking," *IEEE Transactions on Medical Imaging*, vol. 23, no. 1, pp. 130–133, 2004.

[14] E. Meijering, M. Jacob, J. C. F. Sarria, and M. Unser, "A novel approach to neurite tracing in florecence microscopy images," in *Proceedings of the 5th LASTED International Conference on Signal and Image Processing*, pp. 491–495, Honolulu, Hawaii, USA, August 2003.

[15] E. Meijering, M. Jacob, J. C. F. Sarria, P. Steiner, H. Hirling, and M. Unser, "Neurite tracing in fluorescence microscopy images using ridge filtering and graph searching: principles and validation," in *IEEE International Symposium on Biomedical Imaging: Macro to Nano*, vol. 2, pp. 1219–1222, Arlington, Va, USA, April 2004.

[16] C. M. Van Bemmel, M. A. Viergever, and W. J. Niessen, "Semiautomatic segmentation and stenosis quantification of 3D contrast-enhanced MR angiograms of the internal carotid artery," *Magnetic Resonance in Medicine*, vol. 51, no. 4, pp. 753–760, 2004.

[17] A. F. Frangi, W. J. Niessen, P. J. Nederkoorn, J. Bakker, W. P. T. M. Mali, and M. A. Viergever, "Quantitative analysis of vascular morphology from 3D MR angiograms: in vitro and in vivo results," *Magnetic Resonance in Medicine*, vol. 45, no. 2, pp. 311–322, 2001.

[18] W. Cai, F. Dachille, and M. Meissner, "Centerline optimization using vessel quantification model," in *Medical Imaging 2005—Physiology, Function, and Structure from Medical Images*, vol. 5746 of *Proceedings of SPIE*, no. II, pp. 796–803, San Diego, Calif, USA, February 2005.

[19] R. Van Uitert and I. Bitter, "Subvoxel Accurate Skeletons of Volumetric Data Based on Level Sets," submitted.

[20] J. A. Sethian, *Level Set Methods and Fast Marching Methods: Evolving Interfaces in Computational Geometry, Fluid Mechanics, Computer Vision, and Materials Science*, Cambridge University Press, Cambridge, UK, 1999.

[21] S. R. Aylward and E. Bullitt, "Initialization, noise, singularities, and scale in height ridge traversal for tubular object centerline extraction," *IEEE Transactions on Medical Imaging*, vol. 21, no. 2, pp. 61–75, 2002.

[22] C. Kirbas and F. Quek, "A review of vessel extraction techniques and algorithms," Tech. Rep., Vision Interfaces and Systems Laboratory (VISLab), Department of Computer Science and Engineering, Wright State University, Dayton, Ohio, USA, 2002.

[23] A. Huang, G. M. Nielson, A. Razdan, G. E. Farin, D. P. Baluch, and D. G. Capco, "Thin structure segmentation and visualization in three-dimensional biomedical images: a shape-based approach," *IEEE Transactions on Visualization and Computer Graphics*, vol. 12, no. 1, pp. 93–102, 2006.

[24] Y. Sato, C.-F. Westin, A. Bhalerao, et al., "Tissue classification based on 3D local intensity structures for volume rendering," *IEEE Transactions on Visualization and Computer Graphics*, vol. 6, no. 2, pp. 160–180, 2000.

[25] W. Cai, F. Dachille, H. Yoshida, and G. Harris, "Fast, interactive segmentation of vessel in computed-tomographic angiography (CTA) images using selective vesselness-priority region-growing method," in *Radiological Society of North America (RSNA '05)*, McCormick, Chicago, November-December 2005, LPL09-06.

[26] A. Kanitsar, D. Fleischmann, R. Wegenkittl, and E. Grooller, "Diagnostic relevant visualization of vascular structures," in *IEEE Visualization*, Boston, Mass, USA, October-November 2002.

[27] A. Etienne, R. M. Botnar, A. M. C. Van Muiswinkel, P. Boesiger, W. J. Manning, and M. Stuber, ""Soap-Bubble" visualization and quantitative analysis of 3D coronary magnetic resonance angiograms," *Magnetic Resonance in Medicine*, vol. 48, no. 4, pp. 658–666, 2002.

[28] I. Wolf, M. Hastenteufel, I. Wegner, et al., "Curved reformations using the Medical Imaging Interaction Toolkit (MITK)," in *Medical Imaging 2005—Visualization*, vol. 5744 of *Proceedings of SPIE*, no. II, pp. 831–838, San Diego, Calif, USA, February 2005.

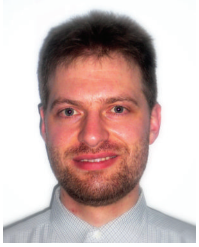

**Ingmar Bitter** received the Ph.D. degree in computer science from the State University of New York at Stony Brook, in 2002. He then led the virtual colonoscopy and 3D medical visualization development as Director of Research and Development at Viatronix Inc, NY. Subsequently, he researched computer-aided detection of colon polyps as well as automated segmentation of blood vessels in CT and MRI as a Staff Scientist at the Diagnostic Radiology Department at the Warren G. Magnuson Clinical Center at the NIH, Bethesda, MD. Currently, he is Lead Engineer, Algorithmics at Claron Technology Inc., where he leads the R&D on efficient and effective CT analysis and interpretation software. He has coauthored over 40 scientific articles and is coinventor on 3 issued and 6 pending patents.

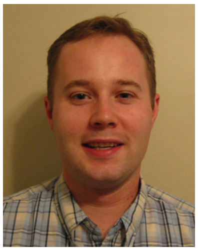

**Robert Van Uitert** received the B.S. degree in computer science from Stanford University, Stanford, CA, in 1998 and the Ph.D. degree in computer science from the University of Utah, Salt Lake City, UT, in 2004. He is currently a Staff Scientist in the Diagnostic Radiology Department of the Clinical Center at the National Institutes of Health. His research interests are in the field of medical image processing and analysis. His particular interests include the use of image processing for computer-aided detection and diagnosis of disease.

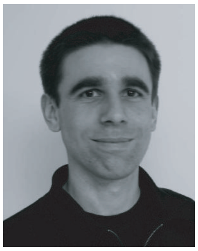

**Ivo Wolf** received the M.S. degree in physics in 1999 and the Ph.D. degree in medical informatics in 2003 from the University of Heidelberg. He is working as a Senior Scientist at the Division of Medical and Biological Informatics at the German Cancer Research Center. His research interests include image segmentation, quantitative image analysis for diagnosis support, software engineering methods to facilitate the transfer of medical image processing techniques into clinically useful software, and navigation systems.

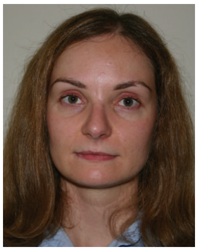

**Efstathia Tzatha** received her B.S. degree in medical imaging from the Technological Institution of Athens, Greece, in 1999 and her M.D. degree from the University of Athens, in 2004. She was a Research Fellow at the National Institutes of Health (2004–2006) and she is currently pursuing further training in medical residency at Georgetown University Hospital. Her interests include assessment of coronary atherosclerosis with MR and molecular imaging.

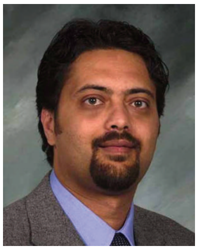

**Ahmed M. Gharib** received his M.B., Ch.B. (Bachelor of Medicine and Surgery) degree from the Faculty of Medicine, the University of Alexandria, Egypt, in November 1993. Following this he served an internship in Internal Medicine at the University of Washington; a residency in Nuclear Medicine and Nuclear Cardiology at the University of Washington; and a residency in Diagnostic Radiology at the University of Louisville before joining the Johns Hopkins Hospital for Cross-Sectional Imaging fellowship for training in Cardiac and body MRI, CT in addition to ultrasound. He then joined the National Institutes of Health as a staff Radiologist, where he focused his research effort on molecular and anatomic imaging of atherosclerosis. He is certified by the American Board of Radiology.

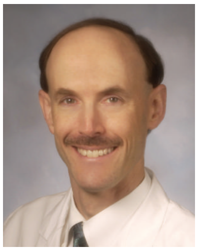

**Ronald Summers** received the B.A. degree in physics and the M.D. and Ph.D. degrees in medicine/anatomy & cell biology from the University of Pennsylvania. He completed a medical internship at the Presbyterian, University of Pennsylvania Hospital, Philadelphia, PA, a radiology residency at the University of Michigan, Ann Arbor, MI, and an MRI fellowship at Duke University, Durham, NC. In 1994, he joined the Diagnostic Radiology Department at the Warren G. Magnuson Clinical Center at the NIH, Bethesda, MD, where he is now a Senior Investigator and Staff Radiologist. He is currently Chief of the Clinical Image Processing Service and directs the Virtual Endoscopy and Computer-Aided Diagnosis (CAD) Laboratory. In 2000, he received the Presidential Early Career Award for Scientists and Engineers, presented by Dr. Neal Lane, President Clinton's science advisor. His research interests include virtual colonoscopy, CAD, and development of large radiologic image databases. His clinical areas of specialty are thoracic and gastrointestinal radiology, and body cross-sectional imaging. He Cochairs the special session on virtual endoscopy at the annual SPIE Medical Imaging Conference. He has coauthored over 100 journal articles, reviews, and conference proceedings articles.

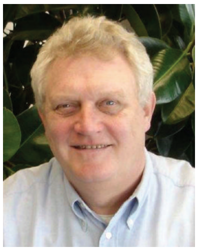

**Hans-Peter Meinzer** is a Scientist at the German Cancer Research Center since 1974, is directing a 3D visualization of 3D tomographies research team since 1983. In this context he works on modeling and simulating tissues and tissue kinetics, neural nets, AI, human perception, cognitive texture analysis, morphology, segmentation, visualization, and medical imaging in general. He obtained at Karlsruhe University an M.S. (physics) and a B.S. (economics) degrees in 1973. At Heidelberg University Meinzer received a doctorate in medical computer science (formal languages) (1983) and habilitation (cell growth simulation) (1987) and is a Professor for medical computer science since 1999. Since 2000 Meinzer is the director of the Department of Medical and Biological Informatics at the German Cancer Research Center in Heidelberg. He is annually co-organizing the German Workshop on Medical Imaging "Bildverarbeitung fuer die Medizin". He won several scientific awards from the German Society of Heart Surgery (1992), the German Society of Pattern Recognition (1993), and the European Commission (1997 and 2003), twice the "European Information Technology Prize." Meinzer founded and directs the Steinbeis-Transferzentrum Medizinische Informatik (STZ-MI) in 1994, a software company specializing in telemedicine and teleradiology. In 2003 he cofounded Chili GmbH, a spin-off company for PACS, teleradiology, web-based image distribution.

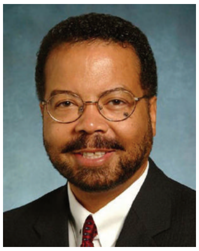

**Roderic Pettigrew** graduated cum laude from Morehouse College with a B.S. degree in physics; earned an M.S. degree in nuclear medicine and engineering from Rensselear Polytechnic Institute; and as Whitaker Harvard-MIT Health Science Scholar a Ph.D. degree in applied radiation physics from the Massachusetts Institute of Technology, and earned an M.D. from the University of Miami, School of Medicine. He did his internship and residency in internal medicine at Emory University and completed a residency in nuclear medicine at the University of California, San Diego. Dr. Pettigrew spent a year as a Clinical Research Scientist with Picker International. In 1985, he joined Emory as a Robert Wood Johnson Foundation Fellow. Dr. Pettigrew, a Member of Phi Beta Kappa, is the Recipient of the Bennie Award (Benjamin E. Mays) for Achievement in 1989, and was named the Most Distinguished Alumnus of the University of Miami, in 1990. He has served as Chairman of the Diagnostic Radiology Study Section, Center for Scientific Review, NIH, and is an elected Fellow of the American Heart Association, the American College of Cardiology, The International Society of Magnetic Resonance in Medicine, The American Institute of Medical and Biological Engineering, and the Biomedical Engineering Society.

